# Supplementary material for: Low Carb and Ketogenic Diets Increase Quality of Life, Physical Performance, Body Composition, and Metabolic Health of Women with Breast Cancer
Source: Nutrients. 2021 Mar 23;13(3):1029. doi: 10.3390/nu13031029 (PMC8004887; doi:10.3390/nu13031029)

Supplemental Material

Methods S1: Description of the multimodal therapy

In Germany, all cancer patients are entitled to attend a three-week inpatient rehabilitation program financed by the German Pension Insurance. Such programs aim to provide patients support in managing the medium- and long-term challenges associated with their condition. The concept of these programs is comprehensive and multiprofessional. Its overarching goal is strengthening patients' self-management competencies by providing them information, skills, and support. Multimodal interventions available comprise medical treatment, physiotherapy, ergotherapy, exercise therapy, psychoeducation, psychological counseling, relaxation treatment and diet counseling. Patients together with their physicians decide on which of these interventions to select, according to patients' needs.

Supplementary Table S1: composition and guidelines for the dietary regimens

Diet type	Composition of calories (total calories ad libitum)	Description	Guidelines	Preferred food items
**SD**	CHO 52-62%;<br>Prot. 16-17%,<br>Fat 28-31%	LowFat diet as recommended<br>standard diet	5 portions fruit or vegetables/day*<br>Whole grain preferred<br>Select healthy fat sources**<br>Reduce sugar and salt	Whole-grain products, potatoes, rice, legumes, all types of fruit (250g/day), salad, all types of vegetable (400g/day), skimmed milk and milk products, fish, sparse meat (preferred poultry) and meat products, plant oils and margarine
**LCD**	CHO 20-30%,<br>Prot. 20-30%,<br>Fat 40-50%	Moderate „fat,<br>protein rich"<br>LowCarb diet	5 portions fruit or vegetables/day*<br>Avoid processed CHO rich/starchy foods<br>Select healthy fat sources**<br>Add plenty protein from plant and animal sources<br>Do not count fat	All types of vegetable, salad, legumes and fruit as staple, plant oils, milk and milk products, low-fat fish, meat and meat products, eggs, nuts, rare full-grain products and potatoes
**KD**	CHO 2-4%,<br>Prot. 16-18%,<br>Fat 80-85%	Ketogenic diet type „modified Atkins diet".<br>Ketogenic Quotient<br>1.6:1 - 2:1	Staple of food must be fat<br>Select healthy fat sources**<br>Add vegetables/salad and fruits low in sugar to every meal***<br>Prefer protein to CHO<br>Calculate ketogenic ratio for every meal	Plant oils (olive, canola, coconut, flaxseed, hempseed), butter, cream, nuts, seeds, olives, avocado, fatty cheese and milk products, Salads, starch free vegetables like broccoli, zucchini, celeriac, parsnip, few berries, eggs, fish, fatty meat, tofu, dark chocolate

ketogenic diet, LCD: low carb diet, SD: standard diet; CHO: carbohydrates; Prot: protein; KD: * all types of fruit, salad and vegetables allowed, 1 portion is at least one hand full, preference should be salad and vegetable over fruit. Natural fruit sources not canned/with added sugar. **as healthy fat sources plant based neutral oils like olive oil, rapeseed oil and coconut oil were recommended and in addition the preference for omega-3 rich plant oils (linseed, hempseed) and avoidance of omega-6 rich fat sources (lard, sunflower-, corn-, safflower oil) were educated.

Supplementary Figure S1

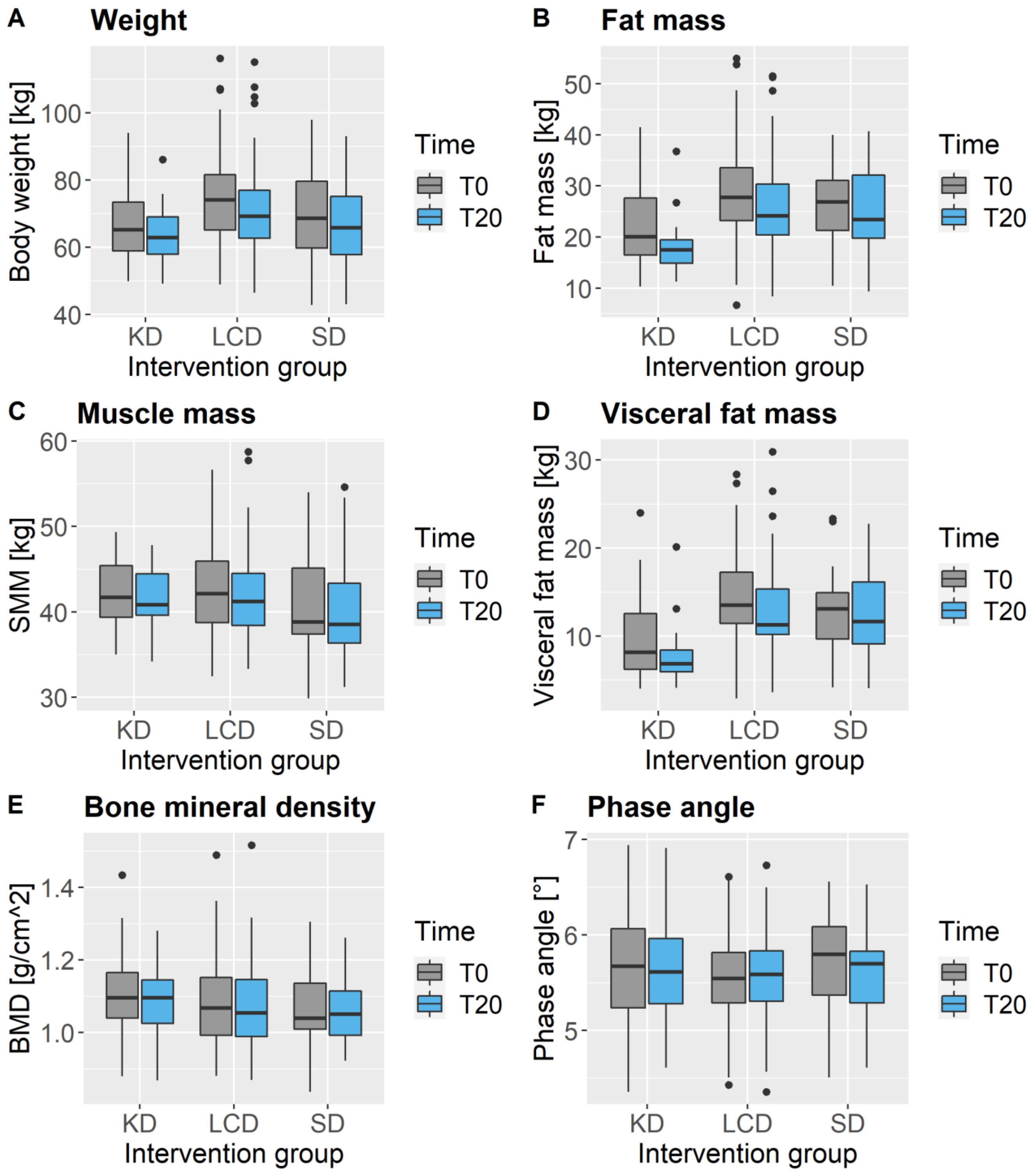

- **Supplementary Figure 1.** Body composition changes from T0 to T20 within each diet group. See Table 3 for further details. KD: ketogenic diet, LCD: low carb diet, SD: standard diet, SMM: sceletal muscle mass, BMD: bone mineral density

Supplementary figure S2-1: Quality of life questionnaire at start (T0) and end (T3) of in house rehabilitation and at the end of intervention (T20): part 1: symptoms

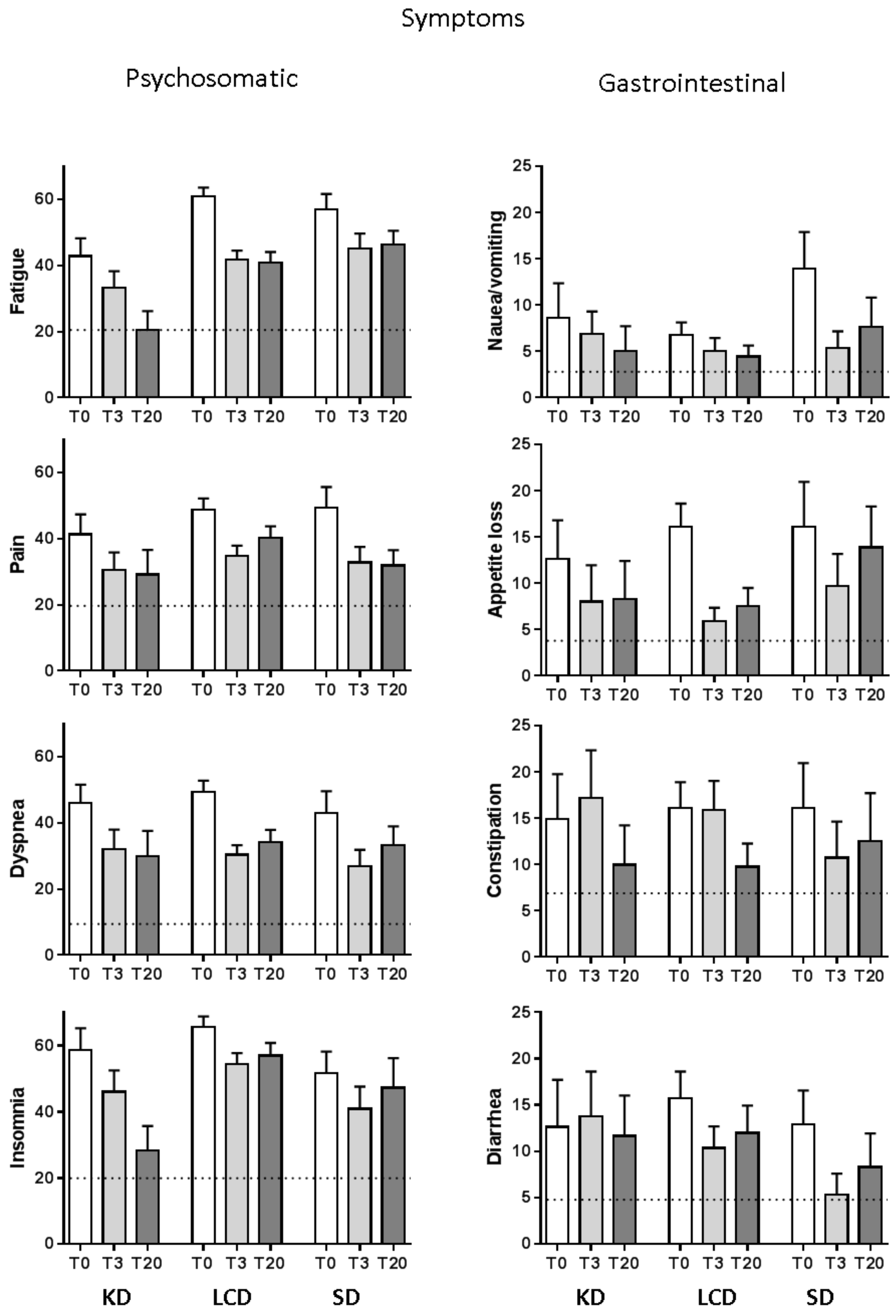

Supplementary figure S2-2: Quality of life questionnaire at start (T0) and end (T3) of in house rehabilitation and at the end of intervention (T20): part 2: functioning scales

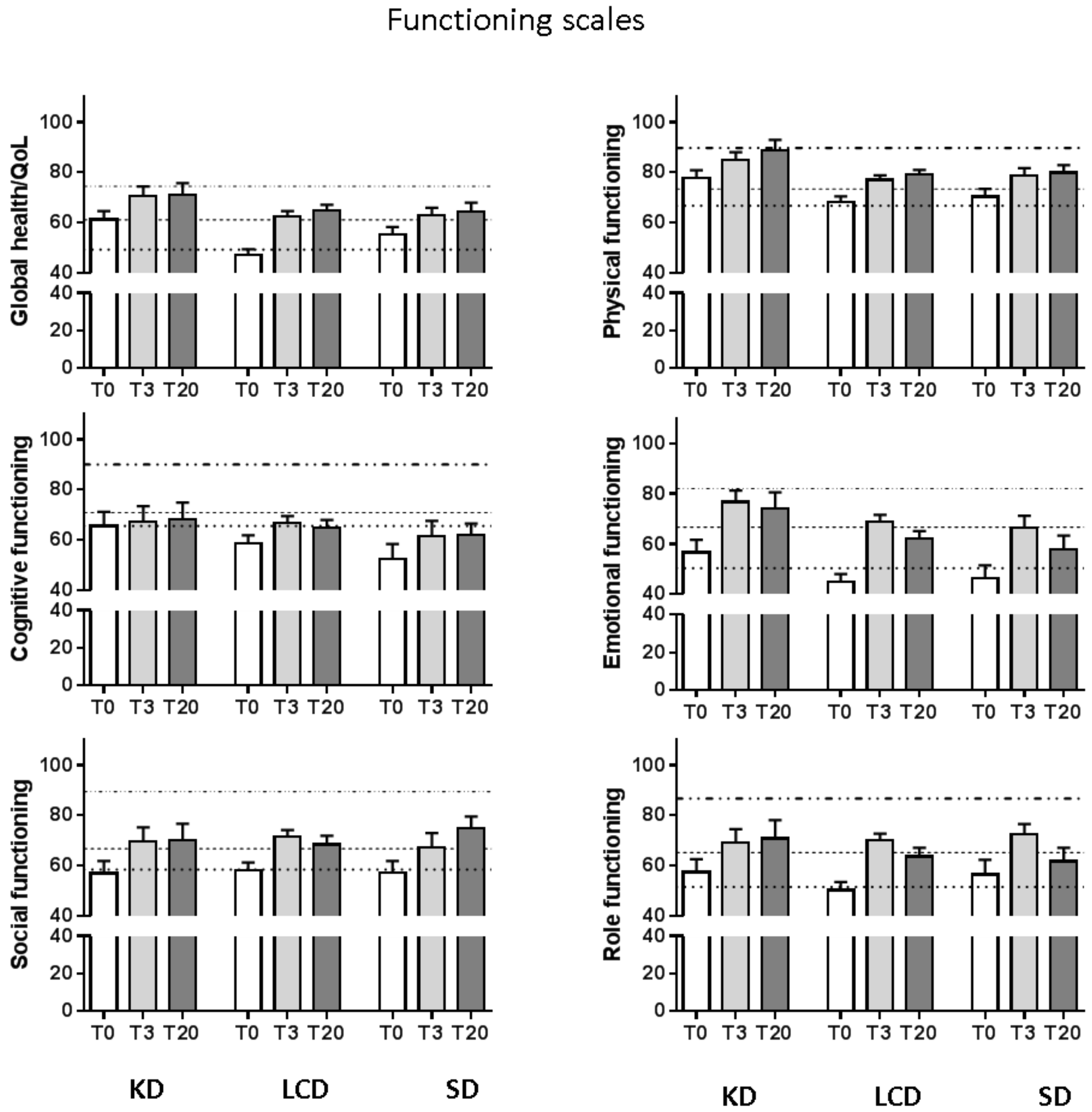

Supplemental Figure S1 shows results of the EORTC-QLQC30 and BR23 questionnaires comparing the aspects of Quality of Life (Qol) before (T0), after 3 weeks of stationary intervention (T3) and after 16 successive weeks of outpatient intervention (T20)

—·-— European reference values in general population (Hinz A, 2014)

----- Mean QoL scores of cancer patients after 3 weeks rehabilitation (Peters E, 2016)

.... Mean QoL scores of cancer patients before rehabilitation (Peters E, 2016)

Supplementary Figure S3-1: Blood parameters at start (T0) and end (T3) of in house rehabilitation and at the end of intervention (T20): part 1: Kidney and liver values. . Dashed lines: upper and lower normal range

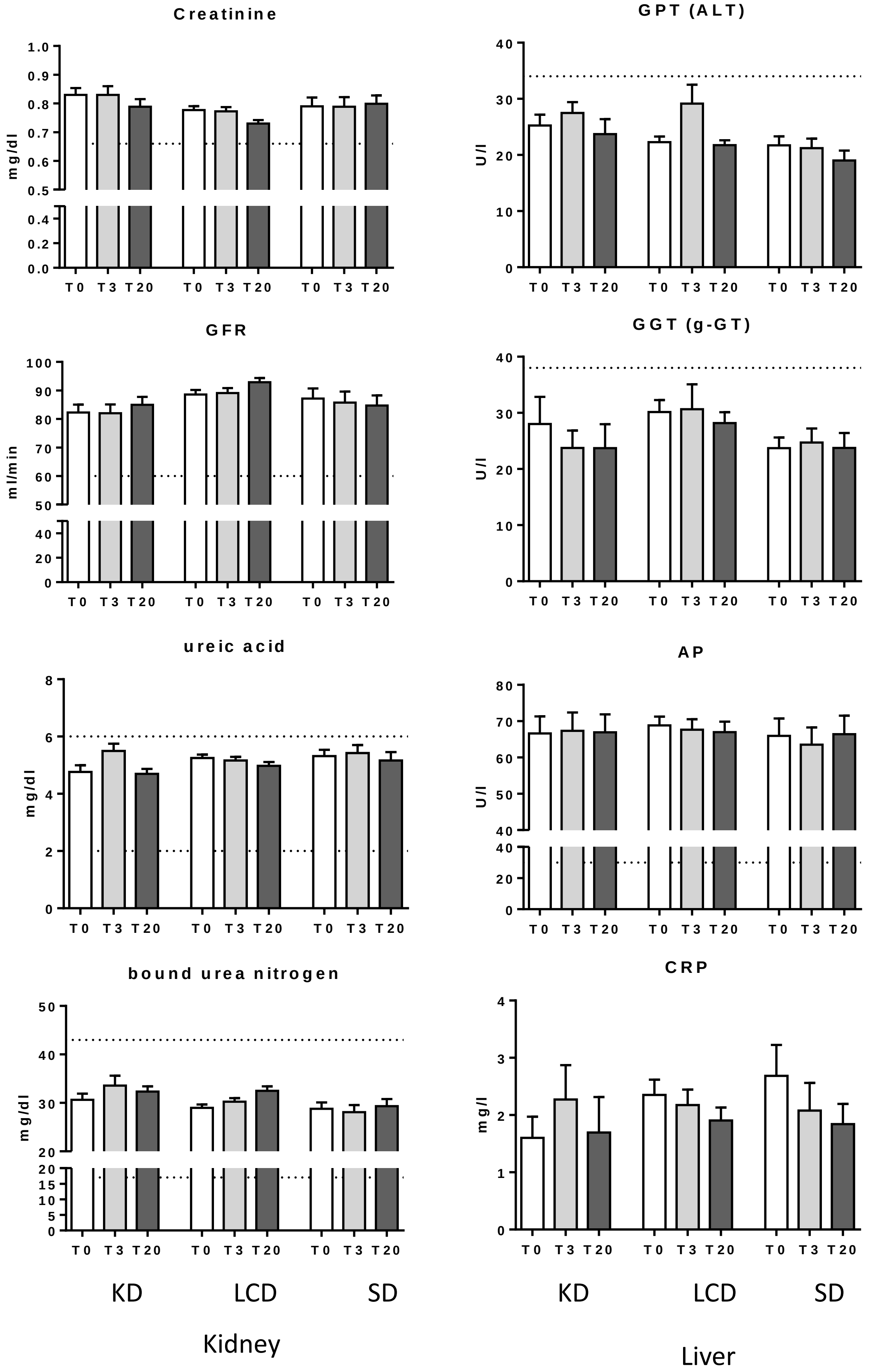

Supplementary Figure S3-2 Blood parameters at start (T0) and end (T3) of in house rehabilitation and at the end of intervention (T20): part 2: blood sugar regulation, protein content and Leucocytes/hemoglobin (HB). . Dashed lines: upper and lower normal range

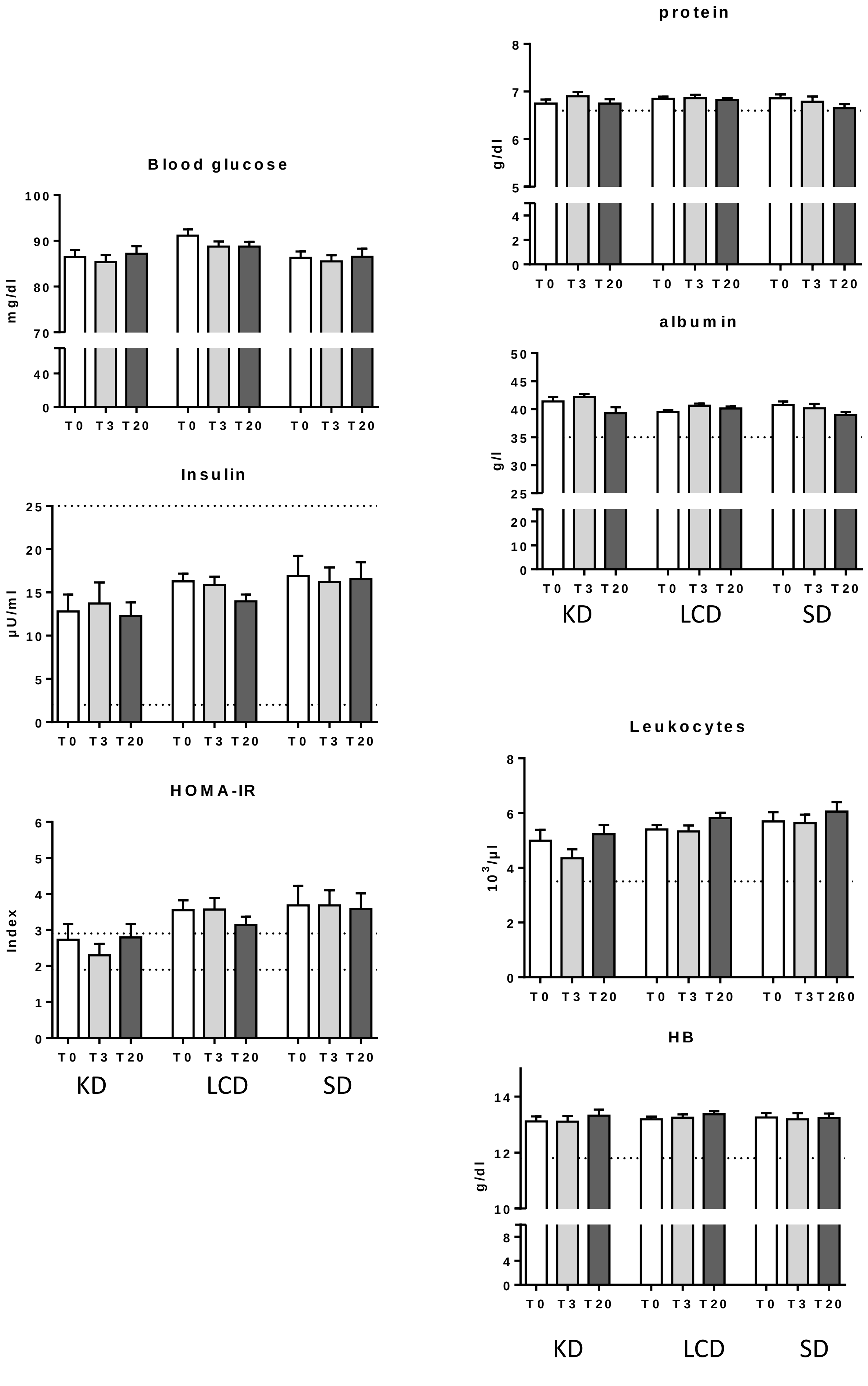

Supplementary Figure S3-2 Blood parameters at start (T0) and end (T3) of in house rehabilitation and at the end of intervention (T20): part 3: blood lipids. Chol: cholesterol, LDL: low-density lipoprotein, HDL: high-density lipoprotein, TG: triglycerides. Dashed line: upper normal range

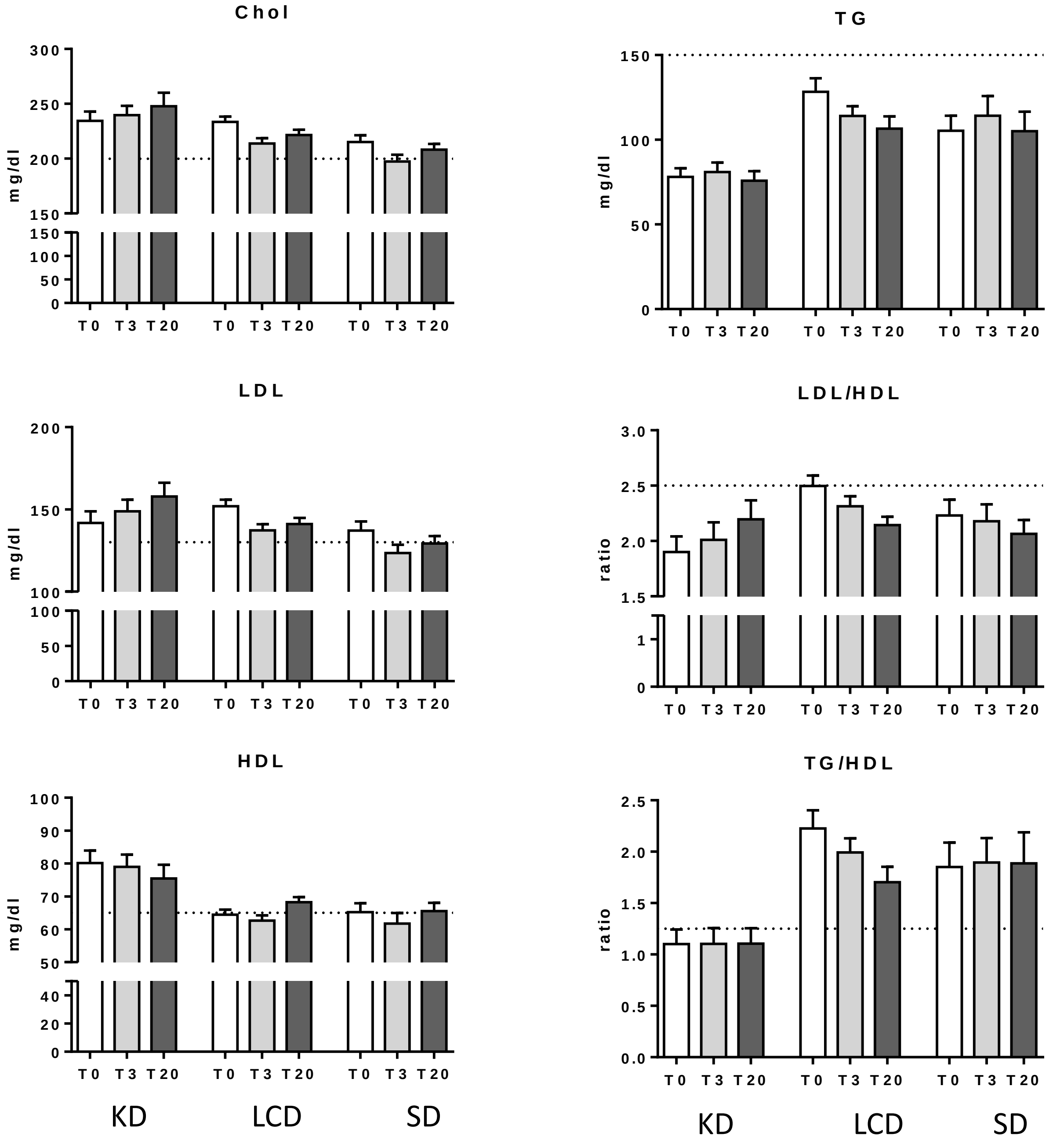

Supplement: Supplementary file 1 [file nutrients-13-01029-s001.pdf]
